# Evaluating a Decade of Surgical Solutions: Pediatric Nasolacrimal Duct Obstruction Treatment Outcomes in Malaysia

**DOI:** 10.7759/cureus.74565

**Published:** 2024-11-27

**Authors:** Chia Yaw Teoh, Kavitha Saravanamuthu, Zulhilmi Abdul Razak, Wan Mariny W Md Kasim

**Affiliations:** 1 Ophthalmology, International Islamic University of Medicine (IIUM), Kuantan, MYS; 2 Ophthalmology/Oculoplasty, Hospital Serdang, Kajang, MYS; 3 Ophthalmology/Glaucoma, International Islamic University of Malaysia (IIUM), Kuantan, MYS

**Keywords:** congenital nasolacrimal duct obstruction, external dacryocystorhinostomy, oculoplastic surgeries, pediatric group, probing, success rate

## Abstract

Objective: This study aims to determine the outcomes of probing and external dacryocystorhinostomy (exDCR) for congenital nasolacrimal duct obstruction (cNLDO) and the factors influencing the success rates in pediatric cNLDO. Design: A retrospective sample collection was conducted at the oculoplastic referral center over 10 years (January 2012 to December 2022) for cNLDO patients who had undergone probing or exDCR. Methodology: Data were retrospectively reviewed for patients aged ≤18 years who underwent probing or exDCR. Variables namely demographics, clinical presentations, indications for surgery, complications, and surgical outcomes were collected for further interpretation. Success was defined as the improvement of symptoms and resolution of clinical signs after at least six months of follow-up. Logistic regression was performed to analyze factors affecting success rates. Results: A total of 109 patients were included (66 exDCR, 43 probing), with nearly similar male-to-female distribution. Common presentations were epiphora and mucous discharge. The majority of cases were simple cNLDO (91% in the probing group, 88% in the exDCR group). Indications for surgery were dacryocystitis, fistula, canaliculitis, and persistent symptoms despite Crigler massage. The success rates were 60.5% for probing and 86.4% for exDCR. Complications varied between procedures and included fistula, tube dislodgement, and granuloma formation. Younger age and the absence of dacryocystitis were significantly associated with higher success rates in probing. Other factors influencing success rates, included simple cNLDO, bilaterality, and the use of endoscopic guidance despite non-significance statistically. Conclusion: The success rates for probing and exDCR were 60.5% and 86.4%, respectively. Statistically significant factors associated with improved outcomes included younger age and the absence of dacryocystitis. Other factors, such as simple cNLDO, bilaterality of disease, and endoscopic guidance, may also contribute to better outcomes, though they were not statistically significant.

## Introduction

Congenital nasolacrimal duct obstruction (cNLDO) is mainly caused by incomplete embryonic canalization of the caudal end of the nasolacrimal duct, affecting 6-20% of newborns [1-4]. It can be classified as simple or complex and is associated with developmental abnormalities, namely trisomy 21, brachiooculofacial syndrome, CHARGE syndrome, and intraoperative findings [1,2]. Simple cases are typically associated with easier, low-resistance probing, whereas complex cases pose greater difficulty requiring expertise in the field [1]. The condition is commonly detected within a few weeks of life coinciding with the timing of tear production, presenting with symptoms such as epiphora, discharge, and matted eyelashes. It can be detected non-invasively using several clinical tests, such as the dye disappearance test (DDT) or regurgitation on pressure over the lacrimal sac (ROPLAS), and further established through probing or endoscopic examination via the nasal approach [1,2]. In some cases, complications can arise such as chronic conjunctivitis, acute dacryocystitis, preseptal, or even orbital cellulitis [1,2]. Management strategies typically depend on the child's age at presentation. Children under one year of age are treated conservatively with lacrimal sac massage or compression for up to 18 months, in the hope that the membrane at the caudal end will break through hydrostatic pressure [1,3,4]. The condition resolves in 96% of cases during the first year [3]. For children over one year of age, treatment often involves probing, though some may require earlier intervention due to dacryocele, acute dacryocystitis, complex cNLDO, or progressively dilated lacrimal sacs. The goal of probing is to manually break the membranous obstruction, and the procedure is more effective with endoscopic guidance. Patency is confirmed using fluorescein dye [1]. The success rate is approximately 97% within the first year and 88% between 12 and 101 months, with endoscopic guidance improving success to 94-97%. Common causes of failure include improper technique, complex cNLDO, and delayed probing [3]. External dacryocystorhinostomy (exDCR) is considered a last resort for cNLDO, usually recommended for children over three years of age or those with complex cNLDO, particularly with congenital lacrimal fistula [1]. The success rate for exDCR ranges from 58-100%, with higher success in simple cNLDO cases (89-97.5%) [1,3]. This study seeks to evaluate the outcomes of probing and exDCR for cNLDO and identify the factors that influence success rates in pediatric cases. In Malaysia, there is a lack of studies regarding the surgical outcomes of cNLDO for both probing and exDCR leading to the conduct of this study and making a comparison to the existing evidence globally.

## Materials and methods

Design, setting, and participants

A retrospective review of electronic records was carried out at an oculoplastic referral center over a 10-year period (January 2012 to December 2022) for patients with cNLDO who had undergone probing or exDCR. *Inclusion Criteria*

Data were retrospectively reviewed for patients aged ≤18 years who underwent probing or exDCR, with at least six months of follow-up. 

Exclusion Criteria

Patients with incomplete data, inadequate follow-up of less than six months, secondary causes of NLDO, inconclusive diagnoses, or incorrect diagnostic coding for cNLDO were excluded.

Definition

Diagnosis of cNLDO in pediatric patients was based on the presence of symptoms (epiphora, discharge, matted eyelashes) with confirmatory tests such as the dye disappearance test or increased tear meniscus height, if applicable to age.

Success was defined as symptom improvement or resolution of clinical signs after at least six months of follow-up. The success rate is defined by the number of successful treatments of cNLDO over the total cases of treated cNLDO.

Statistical analysis and interpretation

Case screening was conducted through manual data collection and filtering. Variables collected included demographics, clinical presentation, indications for surgery, complications, and surgical outcomes.

Data were grouped into probing and exDCR groups. Data is portrayed in descriptive and inferential statistics. Descriptive statistics will include patient demographics, clinical features, and intraoperative factors as well as a success rate comparison between probing and exDCR groups among individual factors enlisted. 

Data was further analyzed using IBM SPSS Statistics, version 29.0.2 (IBM Corp., Armonk, NY) employing multiple logistic regression to identify significant factors influencing success rates in the respective groups. Data were tabulated with descriptive analysis including the patient profile and success rate between probing and exDCR groups. 

The research adhered to the Declaration of Helsinki and followed the Strengthening the Reporting of Observational Studies in Epidemiology (STROBE) reporting guideline.

## Results

A total of 109 children were included in this study (probing n=43, exDCR n=66), with the sample distribution detailed in Table 1. The median age for probing was two years (range of six months to six years), while the median age for exDCR was eight years (range of five to 18 years). The probing group included 27 males and 16 females, while the exDCR group comprised 34 males and 32 females. Most of the children had simple cNLDO (91% probing, 88% exDCR). The history of dacryocystitis was more common in the exDCR group compared to the probing group (42% vs. 21%). The mean duration from diagnosis to surgery was three months (CI 95% 2.77 - 3.68).

**Table 1:** Demographic and clinical sample distribution among probing and exDCR group. exDCR: external dacryocystorhinostomy. *Dacryocystorhinostomy was done without endoscopic guidance. †No repeated exDCR cases in the probing group.

Features	Probing, n (%)	exDCR, n (%)
Gender		
Male	27 (63%)	34 (52%)
Female	16 (37%)	32 (48%)
Categorical		
Simple	39 (91%)	58 (88%)
Complex	4 (9%)	8 (12%)
Laterality		
Unilateral	38 (88%)	57 (86%)
Bilateral	5 (12%)	9 (14%)
Dacryocystitis history		
Present	8 (19%)	27 (41%)
Absent	35 (81%)	38 (59%)
Crilger massage		
Yes	22 (51%)	40 (61%)
No	21 (49%)	26 (39%)
Complication		
Yes	3 (75%)	8 (12%)
No	40 (25%)	58 (88%)
Endoscopic guidance*		
Yes	16 (37%)	
No	27 (63%)	
Repeated exDCR†		
1		4 (6%)
2		2 (3%)
Total	43	66

The primary indications for surgery were persistent symptoms refractory to conservative measures (n=62), followed by dacryocystitis (n=38), fistula (n=8), and canaliculitis (n=1). In the probing group, 35% (n=16) were assisted by nasal endoscopic visualization.

Complications varied between the two procedures. In the probing group, one patient developed a fistula and one had a dislodged tube. In the exDCR group, five patients experienced tube dislodgement, four experienced stent prolapse, and two developed granulomas. The mean follow-up period was 14.5 months. The success rates for probing and exDCR were 60.5% and 86.4%, respectively. Success rates for each clinical factor are presented in Table 2. Children older than one year had lower success rates (41.7% and 52.9%) compared to those younger than one year (85.7%). Simple cNLDO had a better success rate in both the probing (61.5% vs. 50%) and exDCR groups (87.9% vs. 75%). Children without dacryocystitis had double the success rate in the probing group.

**Table 2:** Success rate in each of the clinical factors in both probing and exDCR group. exDCR: external dacryocystorhinostomy. ‡As the distribution of age for probing and exDCR differs, hence the classification was adjusted accordingly. *Dacryocystorhinostomy was done without endoscopic guidance. †No repeated exDCR cases in the probing group.

Features	Probing, %	exDCR, %
Age		
	85.71% (< 1 y.o)	75% (<5 y.o)
	41.7% (1-2 y.o)	93.6% (5-12 y.o)
	52.9% (>2 y.o)	82.6% (>12 y.o)
Gender‡		
Male	60.1%	88.2%
Female	68.8%	84.4%
Categorical		
Simple	61.5%	87.9%
Complex	50%	75%
Laterality		
Unilateral	57.89%	89.5%
Bilateral	80.0%	66.7%
Dacryocystitis history		
Present	33.3%	82.1%
Absent	67.5%	89.5%
Crilger massage		
Yes	60%	76.7%
No	64.5%	87.3%
Complication		
Yes	33.33%	87.50%
No	62.50%	86.21%
Endoscopic guidance*		
Yes	68.8%	
No	55.6%	
Repeated DCR†		
1		66.7%
2		33.3%

Unilateral cNLDO was associated with higher success rates in the probing group. Multiple logistic regression analysis (Tables 3, 4) revealed that the absence of prior dacryocystitis (OR=9.262, p<0.05) and younger age (1-2 years old OR=0.075, p<0.005, >2 years old OR=0.101, p<0.05) were significantly associated with higher success in the probing group, while no other associations were detected in both groups

**Table 3:** Multivariable logistic regression of factors on success rate of probing in cNLDO. A multivariable logistic regression via backward logistic regression (LR) method was performed to study the effects of clinical features on successful treatment of probing in congenital nasolacrimal duct obstruction (cNLDO). The logistic regression model was statistically significant, χ2(4)=18.54 p= 0.05, and explained 47.4% (Nagelkerke R2) of the variance in success treatment of NLDO and the correct prediction rate was about 74.4% of cases for probing group analysis. Results indicated that age (p<0.05), and history of dacryocystitis (p<0.05) are statistically significant factors influencing the success rate of cNLDO treatment. Absence of dacryocystitis history has 9.3 times higher chances to have successful treatment. Age between less than 1 year old is 13.3 times and 9.9 times less likely to have successful NLDO treatment compared to one to two years old or >2 years old.

	Crude			Adjusted		
	OR	95% CI	P-value	OR	95% CI	P-value
Demographic						
Age (adjusted)			0.082			0.044
<1 years old	Ref					
1-2 years old	0.087	0.009-0.867	0.037	0.075	0.009-0.629	0.017
>2 years old	0.091	0.007-1.208	0.069	0.101	0.013-0.793	0.029
Gender	1.536	0.235-10.052	0.654			
Clinical data						
Category	0.00	0.00	1.0			
Duration until surgery			0.910			
1 year	Ref					
1-3 year	1.495	0.178-12.538	0.711			
>3 years	0.900	0.067-12.081	0.937			
Laterality	5.276	0.172-161.479	0.341			
History of dacryocystitis	15.798	1.426-175.007	0.024	9.262	1.182-72.567	0.034
Crigler massage	0.230	0.027-1.967	0.180			
Operative						
Endoscopic guidance	2.063	0.386-11.017	0.397			
Complication	0.00	0.00	1.0			

**Table 4:** Multivariable logistic regression of factors on success rate of exDCR on cNLDO. A multivariable logistic regression via the backward logistic regression (LR) method was performed to study the effects of clinical features on the successful treatment of external dacryocystorhinostomy (exDCR) in congenital nasolacrimal duct obstruction (cNLDO). The logistic regression model was statistically significant, χ2(4)=6.333 p=<0.001, and explained 26.7% (Nagelkerke R2) of the variance in success treatment of NLDO and the correct prediction rate was about 89.4% of cases for probing group analysis. No statistically significant association was found in the exDCR group.

	Crude		
	OR	95% CI	P-value
Demographic			
Age (adjusted)			0.797
<6 years old	ref		
6-12 years old	1.199	0.084-17.076	0.893
>12 years old	0.542	0.061-4.823	0.583
Gender	0.366	0.055-2.430	0.298
Clinical data			
Category	0.145	0.013-1.670	0.121
Laterality	0.085	0.007-1.045	0.054
Duration until surgery	0.445	0.095-2.083	0.304
History of dacryocystitis	0.328	0.054-2.002	0.227
Crigler massage	0.247	0.010-5.848	0.387
Operative			
Complications	1.224	0.083-18.069	0.883

## Discussion

External dacryocystorhinostomy (exDCR) is the gold standard for treating nasolacrimal duct obstruction (NLDO) due to its high success rate and manageable complications compared to probing. However, it is not often considered the first-line treatment due to worries about compromising osseous growth and invasive, which has led to a shift towards less invasive and less destructive treatments [2]. This paradigm shift has changed the focus of research regarding the timing of treatment in relation to its efficacy at different ages. In line with most findings, exDCR demonstrated a higher success rate compared to probing in the treatment of congenital NLDO, regardless of age. The success rates of probing (85%, range 61-90%) and exDCR (91%, range 88-100%) in this study were comparable to those reported in previous studies [4-21]. Two key factors emerged from this study: patient age and a history of acute dacryocystitis. Younger patients, especially those undergoing probing, were associated with better success rates, consistent with most existing studies, which show that success rates decline after two years of age [2-16]. This decline may be due to the increased likelihood of complex NLDO, infection, and the development of fibrosis in older children, all of which make probing more difficult and less successful [4-6]. Acute dacryocystitis has also been linked to fibrosis after resolution, possibly by altering the structure of the lacrimal drainage system hence directly impacting the success of probing [22-23]. This is further reflected in the variable success rates (55%-100%) reported by few studies, with chronicity and mucoid discharge associated with lower success rates. Although not statistically significant, simple cNLDO cases had higher success rates compared to complex cases, similar to findings from previous studies. This may be due to the formation of osseous membranes in complex cases, making probing difficult with higher failure rates [4,7-10]. Bilateral cNLDO was associated with better outcomes than unilateral cases, though one study reported the opposite, leaving its influence on success rates controversial [7]. Furthermore, endoscopic guidance during probing slightly improved success rates, although this finding was not statistically significant. Numerous studies have shown the effectiveness of endoscopic probing for cNLDO, which is further supported by systematic reviews despite of bias addressed by the review [24-26]. Notably, the success rate decreased with repeated dacryocystorhinostomy, though this may be biased due to the small number of patients analyzed. The same applies to reduced success rates in cases with complications during probing. When comparing multiple studies with current results, the success of probing appears to be influenced by patient age at presentation and a history of dacryocystitis. Other plausible factors, such as complex cNLDO and the use of endoscopic guidance, are consistent with previous studies. Interestingly, this study shows there is a possible high similarity between the Asian population and the global. Nevertheless, further studies are needed to establish consistency in treatment outcomes within Asian populations. This study is limited by its relatively small sample size and the exclusion of children with cNLDO who underwent conservative management. Including these patients in further study would help provide a better comprehensive comparison between conservative and early surgical approaches, in particular probing, which could lead to a reevaluation of the timing of interventions. It would also aid in identifying children who may benefit from earlier probing, thus optimizing the chances of successful treatment for cNLDO.

## Conclusions

The success rates of probing and exDCR in this study were comparable withthe majority of studies. Significant factors associated with improved surgical outcomes included patient age at presentation and a history of dacryocystitis. Additional factors that may improve surgical outcomes include simple cNLDO, bilaterality of the disease, and the use of endoscopic guidance.
